# Retreatment with a flow diverter for recurrent blood blister-like aneurysms after embolization: A single-center case series

**DOI:** 10.3389/fneur.2022.1009914

**Published:** 2022-10-06

**Authors:** Yazhou Yan, Shijie Zhu, Hao Yao, Yina Wu, Zhiwen Lu, Yibin Fang, Kaijun Zhao, Qinghai Huang

**Affiliations:** ^1^Stroke Center, Changhai Hospital Affiliated to the Naval Military Medical University, Shanghai, China; ^2^Department of Neurosurgery, 971 Hospital of People's Liberation Army (PLA), Qingdao, China; ^3^Department of Neurosurgery, Jinjiang City Hospital, Quanzhou, China

**Keywords:** intracranial aneurysm, recurrent blood blister-like aneurysms, endovascular treatment, flow diverter, stent-assisted coiling

## Abstract

**Background and purpose:**

Treatment of blood blister-like aneurysms (BBAs) has been a significant challenge mainly due to their high recurrence rate even after stent-assisted coiling (SAC) embolization. This study aims to evaluate the safety and efficacy of treating recurrent BBAs after SAC with a flow diverter (FD).

**Methods:**

A retrospective series of patients with recurrent BBAs who underwent the retreatment with the FD from June 2018 to December 2021 was included to analyze perioperative safety and immediate postoperative and follow-up outcomes.

**Results:**

The study enrolled 13 patients with recurrent BBAs previously treated with SAC. Within previous stents, an FD was deployed for retreatment, including eight Tubridge FDs and five PEDs. The time interval between initial treatment and FD implantation was 14–90 days. A total of 11 cases were treated with a single FD alone; two cases were treated with further endovascular coiling embolization, followed by FD implantation. The angiographic follow-up (6–12 months) was available in 12 patients, and all 12 recurrent BBAs were completely occluded. No perioperative complication was detected, and no rebleeding was found during the clinical follow-up (6–36 months).

**Conclusion:**

The use of the FD to manage recurrent BBAs after SAC is technically feasible, safe, and effective. The key to the success of the procedure is to ensure that the FD stent is fully open and has good apposition with the previously implanted stent.

## Introduction

Blood blister-like aneurysms (BBAs) normally refer to small, bleb-like and unidentifiable neck lesions without the internal elastic lamina, vascular intima, and media. Sometimes appearing as only a fragile fibrous layer usually at the anterior or anteromedial wall of the supraclinoid segment of the intracranial carotid artery (ICA), BBA makes up 0.3–1.7% of all intracranial aneurysms and 0.5–2% of ruptured aneurysms (1). Due to its fragile state and difficult morphology, treatments including surgical clipping and endovascular treatment of such lesions have proven to be intractable (2). The overlapped stent-assisted coiling (SAC) technique for the treatment of BBAs may reduce the recurrence rate to an extent, but recurrence rates still range from 5.6 to 22.9% (3, 4). The optimal treatment of recurrent BBAs, including the timing, antiplatelet drugs, and treatment strategies (SAC, flow diverter, etc.), has not yet been determined.

A flow diverter (FD) is used in the treatment of BBAs and is associated with more favorable angiographic outcomes but also with complications and clinical outcomes, compared with SAC (5). However, for previously stented aneurysms, FD treatment had been reported to be less effective and prone to complications (6). Whether the effectiveness of FD implantation for recurrent BBAs after SAC is truly as poor as that of saccular aneurysms needs further research. Hence, we would like to report our initial experience in the retreatment of recurrent BBAs with FDs.

## Materials and methods

### Study design and patients

We have reviewed all patients with recurrent BBAs who were previously treated with SAC and received the retreatment with FDs at our institution between June 2018 and December 2021. A total of 13 patients with 13 recurrent BBAs were identified. All of these patients were initially treated at other hospitals. The first angiographic follow-up was performed within 2 weeks to 1 month after the initial treatment. The patients with major recurrence were referred to our hospital for further treatment. In total, two neurointerventionists assessed the surgical notes, medical charts, and radiologic images of the patients. Moreover, the patients' demographics, aneurysm characteristics, complications, and follow-up data were evaluated.

### Procedure and perioperative medication

All procedures were conducted under general anesthesia *via* a transfemoral approach using biplane angiographic equipment. Heparin (4,000–5,000 U) was intravenously infused after the femoral sheath was placed, with the goal of maintaining the activated clotting time at 2.0–2.5 times as baseline during the procedure. A 6-F guiding catheter was introduced into the distal ICA. On the basis of the images generated from the reconstruction, the working projections were chosen for the procedure. Over a J-shaped tip microwire, a T-track microcatheter (0.029-inch diameter, MicroPort, Shanghai, China) or Marksman microcatheter (Medtronic, Irvine, CA) was passed through the previous stents to the M2 segment of the middle cerebral artery. Then, different position projections or VasoCT/XperCT were performed to ensure that the microcatheter was positioned completely within the previous stents. According to the measured data and the size of previous stents, an appropriate FD stent was selected. The FD was navigated and deployed across the dissecting segment using a standard push-and-pull technique in all cases. After deployment, angiography was used to document the correct expansion of the device. All patients underwent postoperative CT straight to detect any possible intracranial hemorrhage.

Dual antiplatelet drugs (100 mg/day aspirin plus 75 mg/day clopidogrel or 90 mg two times/day ticagrelor) were administered for at least 3 days before the procedure. All patients were administered aspirin (100 mg/day) and clopidogrel (75 mg/day) postoperatively. Postoperative DSA evaluation was performed at 2 weeks to 1 month, and the antiplatelet protocol was adjusted according to the angiographic results. For patients with aneurysm occlusion, they were administered with aspirin (100 mg/day) and clopidogrel (75 mg/day) for 6 weeks, followed by aspirin alone (100 mg/day) indefinitely.

### Angiographic assessment and angiographic and clinical follow-up

The immediate angiographic results of BBAs after initial SAC were assessed using the Raymond scale (7): Raymond 1 shows complete occlusion, Raymond 2 residual neck, and Raymond 3 residual aneurysm. The immediate angiographic results of recurrent BBAs after the retreatment with the FD and follow-up outcomes were classified into five categories according to the Kamran–Byrne scale (8): Grade 0, which denotes no change; grade 1 (residual contrast filling >50% of the pretreatment aneurysm volume), grade 2 (residual contrast filling <50% of the pretreatment aneurysm volume), grade 3 (residual filling confined to the neck region), and grade 4 (complete obliteration). Recurrence was defined as increased contrast material filling the aneurysm sac compared with the immediate degree of embolization. The angiographic follow-up was generally performed at 1, 3, and 6 month post-treatment using DSA, and then yearly thereafter with DSA/CTA. The clinical outcome was assessed using the modified Rankin Scale (mRS) at the latest follow-up.

## Results

### Baseline characteristics

A total of 13 patients (all female) with 13 BBAs were identified. These patients had a mean age of 48.9 (ranging from 42 to 55) years. All patients had a prior history of subarachnoid hemorrhage (Hunt–Hess grade from 1 to 3). All 13 BBAs were located at the supraclinoid segment (eight located at the C6 segment and five at the C7 segment) of the ICA, with 11 at the right ICA, while other two at the left. Overall, four patients were treated with a single LVIS stent (MicroVention Terumo, California, USA) or Enterprise stent (Codman&Shurtleff, Massachusetts, USA), four patients were treated with overlapped LVIS stents, four patients were treated with overlapped LVIS combined with Enterprise stents, and one patient with overlapped LVIS and LEO Plus stents (Balt, Montmorency, France). Immediate angiographic results after initial SAC showed Raymond 1 in three aneurysms, Raymond 2 in six aneurysms, and Raymond 3 in four aneurysms. After the initial treatment, no patient suffered from rebleeding. All recurrent BBAs were confirmed by DSA and referred to our hospital for further treatment, and the time interval between initial treatment and the first angiography follow-up was 14–90 days. The data are summarized in Table 1.

**Table 1:** Clinical data of all the patients.

**Variable**	Values^*^
Age (years)	48.9 (42–55)
Initial H-H Grade	
1	2
2	6
3	5
4	–
5	–
Location	
Right ICA	11 (84.6%)
Lift ICA	2 (15.4%)
Initial treatment strategy	
Single LVIS or Enterprise+Coils	4
Overlapped LVIS+Coils	4
LVIS+Enterprise+Coils	4
LVS+LEO plus+Coils	1
Initial Raymond grade	
1	3
2	6
3	4
Initial angiographic FU (days)	49.3 (14–90)
FD model	
Tubridge	8
Pipeline embolization devices	5
Postoperative K-B scale	
0	7
1	3
2	1
3	2
Angiographic follow-up (months)	7.6 (1–12)
Angiographic follow-up K-B scale	
0	13
1	–
2	–
3	–

H-H Grade, Hunt-Hess grade; ICA, intracranial carotid artery; FU, follow up; FD, flow diverter; K-B scale, Kamran-Byrne scale.

* Values are mean (minimum-maximum value) or number of patients (percentage).

### Technical success and immediate results

The FD deployment was successful among all 13 patients, including eight Tubridge FDs (MicroPort Medical Company, Shanghai, China) and five pipeline embolization devices (PEDs; Medtronic, Irvine, CA). In all, 11 recurrent BBAs were treated by a single Tubridge FD or PED alone. Due to the obvious recurrence of the BBAs and the large size of the aneurysms, the remaining two aneurysms were first treated with further intrasaccular coiling, followed by Tubridge FD deployment. Based on the size of recurrent BBA, four and three coils were deployed during the procedure, respectively. All 13 FD stents were documented as being good wall appositions. In four patients, the FD was completely located inside the previous LVIS stent (not beyond the distal and proximal ends), and for the other nine patients, the FD stents were deployed to span the entire length of the LVIS or Enterprise stent. According to the Kamran–Byrne scale, immediate postoperative angiograms showed grade 0 in seven aneurysms, grade 1 in three aneurysms, grade 2 in one aneurysms, and Grade 3 in two aneurysms, following the retreatment, and the parent arteries and the covered branches by FD stents were all patent (Table 1).

### Angiographic and clinical follow-up

**Figure 1:**
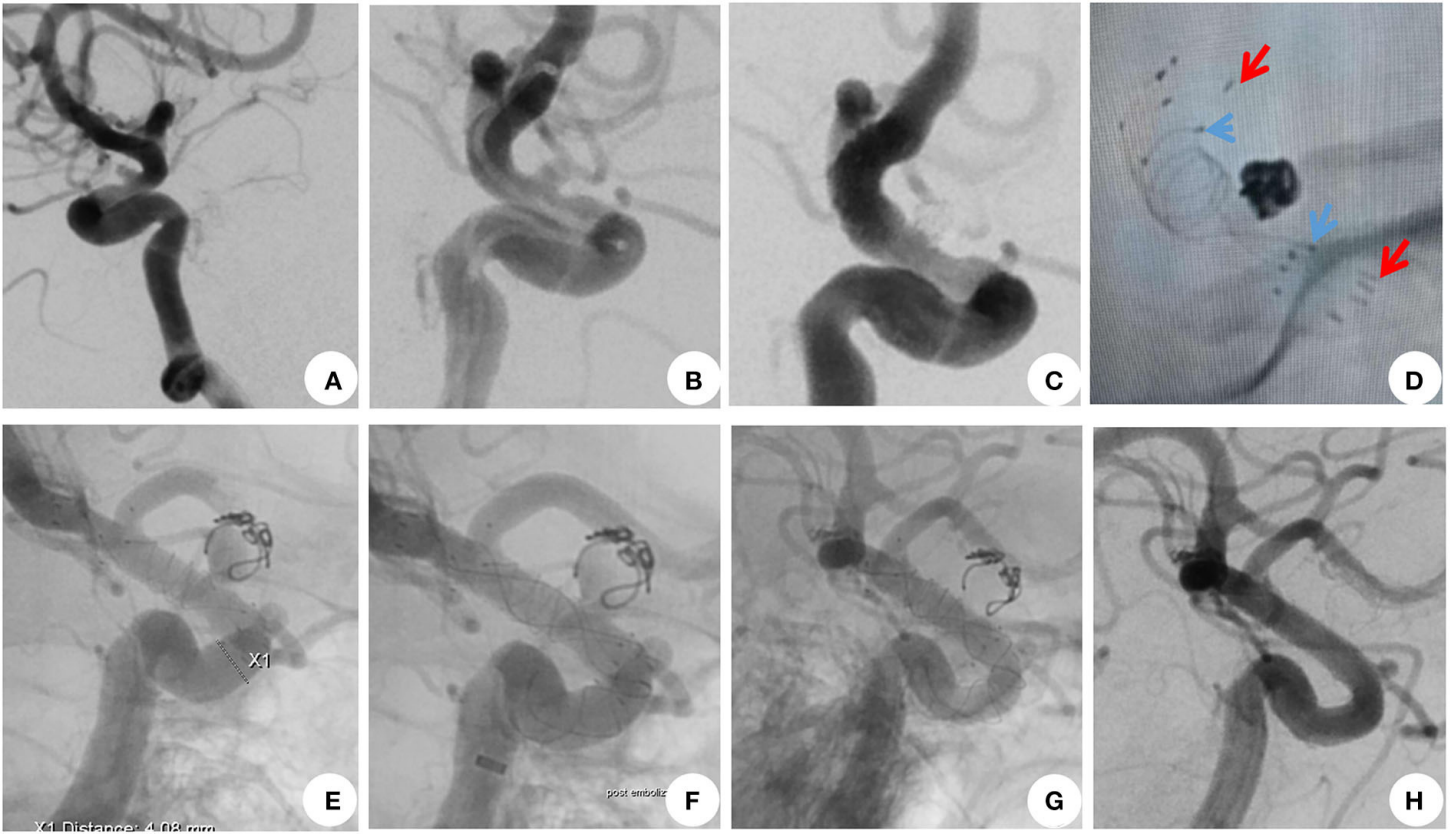
A 44-year-old woman with recurrent blood blister-like aneurysms (BBAs). **(A,B)** DSA showed a BBA located at the left supraclinoid segment of the intracranial carotid artery. **(C,D)** BBA was treated with overlapped LVIS and Enterprise stent-assisted coiling. The immediate postoperative angiogram showed the complete occlusion of the BBA. **(E)** At 1 month after the initial treatment, DSA showed that the BBA had recurred, and the coils were obviously compressed and dispositioned. **(F)** Recurrent BBAs were treated with a 4.0 × 25-mm Tubridge, which covered the aneurysm neck, and the stent opened well. **(G,H)** A 1-month follow-up angiography revealed complete occlusion of BBA and patency of the left intracranial carotid artery. The arrowheads are the ends of the LVIS and Enterprise stents.

Of all 13 patients, the angiographic follow-up (1–12 months) was available for 12 patients. It has showed that aneurysms of 10 patients completely occluded 1 month after the retreatment (Figure 1), whereas two aneurysms remained stable at the 1-month follow-up check. Dual antiplatelet therapies have therefore been changed to aspirin alone, and these aneurysms were completely occluded 2 months later. CTA showed that the two aneurysms had no sign of recurrence 1 year after the procedure. The parent arteries were all patent without any in-stent stenosis. The clinical follow-up at 6–36 months among all patients showed neither hemorrhagic nor thromboembolic events. The mRS score was 0 in all patients at the latest follow-up (Table 1).

## Discussion

BBAs are rare vascular lesions and therapeutic challenges. Although several surgical strategies or endovascular treatments, including SAC or FD, have been proposed, there is no consensus on the optimal treatment for BBAs (9, 10). With fragile walls and an indistinct neck, surgeons are more likely to opt for a conservative strategy to prevent intraoperative rupture and result in low coil packing density. Therefore, SAC for the treatment of BBAs still has a high level of aneurysm rupture, recurrence, and rebleeding risk. A previous study has shown that complete occlusion rates associated with single, 2, and ≥3 stents were 42.9, 78.4, and 88.2%, respectively (3). Since the application of an FD in refractory aneurysms has achieved satisfactory results, it has also been tried in BBAs, which was first reported by Çinar et al. (11) in 2013. Of all seven BBAs, two were managed initially by other endovascular treatment options. Since then, more and more studies have reported the results of FDs in BBAs treatment. A meta-analysis confirmed that compared with other vascular reconstruction strategies, FD treatment has a higher rate of long-term complete occlusion and a lower rate of aneurysm recanalization but similar complications and clinical outcomes (5). Therefore, an FD can be used as an important treatment modality, even in the acute stage of ruptured BBAs. Because FD stents are not always available in some hospitals, SAC is still the main method, despite of its potential risk of recurrence.

Previously stented aneurysms with the retreatment with FDs turned out to be less effective and led to more complications. The occlusion rate reached 40.9–75% for aneurysms, while the complication rate could be as high as 16.7% (12–14). These unfavorable results might be related to the technique of deploying FDs within the previously deployed stent (15). First, the FD device may not be fully positioned within the previously implanted stent because the microwire may cross the previous stent through the cells in an “in–out–in” manner, and as the result, the FD was caught on the prior stent struts. However, in this study, of all 13 recurrent BBAs, 12 were treated with LVIS stents which had a relatively small cell size. When a microwire was advanced through the previous stent, rotating and pushing a J-shaped-tip microwire has proven to be an effective technique, which could prevent the microwire from going through the cells of previous stents. Second, the previous coil mass might interrupt the visibility of the FD. Fortunately, previously treated BBAs tended to have less impact on the visibility of FD stents because of their smaller size and less coil packing. When the wall position of the FD could not be determined due to the surrounding coil mass, balloon angioplasty was performed. Moreover, all 13 BBAs located at the supraclinoid segment of the ICA without the influence of the bony structures of the cranial base, and full openings of all the FDs were able to be detected.

Indeed, one of the important factors affecting the aneurysm occlusion rate was the wall apposition of the FD device (16). Poor vessel wall apposition of the FD would lead to not only acute thrombosis but also an endoleak between the FD and the vessel wall, which might be the reason for persistent aneurysm filling and become an obstacle to neointima formation (17). Adequate opening and good wall apposition of FDs were critical for therapeutic efficiency, especially in the lesions previously treated with stents. It may be a good strategy to place the proximal and distal ends of the FD device beyond the previous stent, at the normal parent artery. This would allow the potentially unidentifiable gap between the parent artery and the previous stent to become a semi-enclosed space, which may gradually disappear. However, if the previously deployed stents were detected to have good wall apposition, in order to reduce the impact of FDs on normal branches and the difficulty of FD opening in vessels with tortuous curves or stenosis, selecting a shorter FD to cover only the distal and proximal ends of BBA neck could also achieve an ideal result. In addition, an intracranial covered stent, which could immediately isolate aneurysms from parent arteries, has also been used in the treatment of BBAs. Qi et al. (18) reported eight BBAs, including five recurrent BBAs treated with Willis covered stent. Follow-up results showed that all patients were in good condition without recurrence, while one patient developed delayed bleeding. However, because of the properties of this stent, its operation was relatively complex, and navigating the covered stent into the paraclinoid ICA was technically challenging and sometimes dangerous. Also, its impact on covered side branches could not be ignored. Further evaluation of the safety and efficiency of the covered stents is needed.

In conclusion, the treatment of BBAs remains technically challenging, with a high recurrence rate even after SAC embolization. In selected patients, the application of FDs for the retreatment of recurrent BBAs seems to be safe and effective. The key to the success of the procedure was to ensure that the FD stent had a full opening and had good apposition with the previously implanted stent. This study also has some major limitations, including the small sample size, the short angiographic follow-up period, single-center nature, retrospective design, and lack of comparison with a control group of patients—for instance, patients who were treated with SAC but did not require retreatment, which allowed higher durability of treatment.

## Data availability statement

The original contributions presented in the study are included in the article/supplementary material, further inquiries can be directed to the corresponding author.

## Ethics statement

The studies involving human participants were reviewed and approved by the Institutional Review Board of Changhai Hospital. The patients/participants provided their written informed consent to participate in this study.

## Author contributions

YY and QH: conception and design. YY, SZ, and HY: data collection and statistical analysis. YY, SZ, HY, YW, and ZL: data analysis, interpretation, and drafting of the manuscript. YF and KZ: editing. QH: review and approval of the final version on behalf of all authors. YY: administrative, technical, and material support. All authors contributed to the article and approved the submitted version.

## Funding

The study was supported by the National Key R&D Program of China (2016YFC1300700), Natural Science Foundation of China (No. 81971089), and 234 Discipline Peak Climbing Program (2020YXK021).

## Conflict of interest

The authors declare that the research was conducted in the absence of any commercial or financial relationships that could be construed as a potential conflict of interest.

## Publisher's note

All claims expressed in this article are solely those of the authors and do not necessarily represent those of their affiliated organizations, or those of the publisher, the editors and the reviewers. Any product that may be evaluated in this article, or claim that may be made by its manufacturer, is not guaranteed or endorsed by the publisher.
